# Pretreatment of baicalin and wogonoside with glycoside hydrolase: A promising approach to enhance anticancer potential

**DOI:** 10.3892/or.2013.2726

**Published:** 2013-09-09

**Authors:** CHUNHAO YU, ZHIYU ZHANG, HAIJIANG ZHANG, ZHONG ZHEN, TYLER CALWAY, YUNWEI WANG, CHUN-SU YUAN, CHONG-ZHI WANG

**Affiliations:** 1 School of Life Science and Chemical Engineering, Huaiyin Institute of Technology, Jiangsu 223003, P.R. China; 2 Tang Center for Herbal Medicine Research, University of Chicago, Chicago, IL 60637, USA; 3 Department of Anesthesia and Critical Care, University of Chicago, Chicago, IL 60637, USA; 4 Department of Medicine, University of Chicago, Chicago, IL 60637, USA; 5 Committee on Clinical Pharmacology and Pharmacogenomics, University of Chicago, Chicago, IL 60637, USA

**Keywords:** glycoside hydrolase, cellulase, flavonoid glycosides, aglycons, *Scutellaria baicalensis*, cancer chemoprevention, cell cycle, apoptosis

## Abstract

Previous phytochemical studies showed that the major flavonoids in *Scutellaria baicalensis* are baicalin, baicalein, wogonoside and wogonin. The two glycosides (baicalin and wogonoside) can be transformed into their aglycons (baicalein and wogonin), which possess positive anticancer potential. In this study, we used glycosidase to catalyze flavonoids in *S. baicalensis* to enhance the herb’s anticancer activities. Our HPLC data showed that, using the optimized conditions obtained in our experiments (20 U/g of cellulase, 50ºC, pH 4.8 and treatment for 8 h), there was a marked transformation from the two glycosides to their aglycons. The anticancer activity was subsequently evaluated using a series of *S. baicalensis* extracts in which variable lengths of glycosidase treatment time were used. Combining analytical and bioassay results, we observed that the higher the aglycon content, the stronger the antiproliferation effects. Compared to the untransformed control, 8 h of glycosidase catalyzing significantly increased antiproliferative activity on human colorectal and breast cancer cells, and its cancer cell growth inhibition is, in part, mediated by cell cycle arrest at the S-phase and induction of apoptosis. Data from this study suggest that using glycosidase to catalyze *S. baicalensis* offers a promising approach to increase its anticancer activity.

## Introduction

Traditional Chinese medicine (TCM) has played a critical role in the promotion of health, prevention of disease, and treatment of illnesses for thousands of years in China and other Asian countries (1,2). Many commonly used Chinese herbal medicines are listed in the Chinese Pharmacopoeia. The dried roots of *Scutellaria baicalensis* Georgi (Labiate) or *S. baicalensis* is one of the most widely used herbs, and this herb is utilized as a key ingredient in many TCM formulations (3).

**Figure 1:**
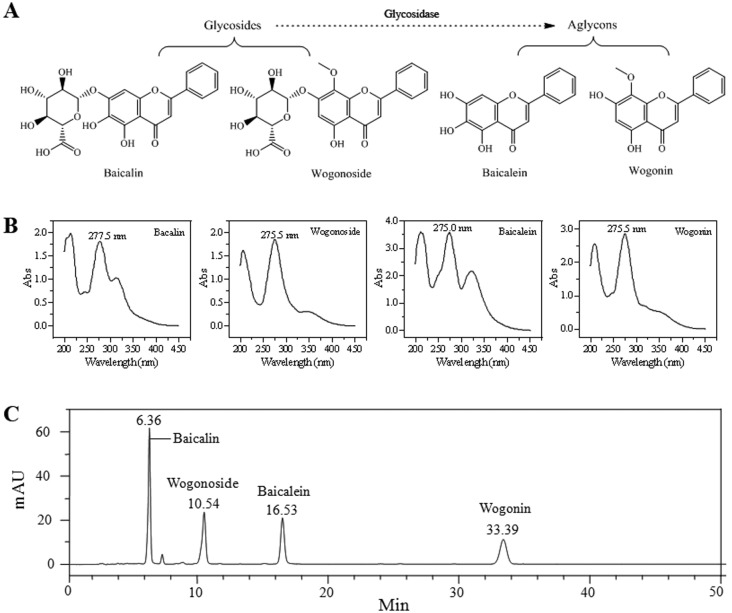
Spectral and chromatographic analysis of *S. baicalensis* compounds. (A) Chemical structures of four major flavonoids from *S. baicalensis*, i.e. baicalin, wogonoside, baicalein and wogonin. (B) Ultraviolet spectroscopy, and (C) HPLC chromatogram the four flavonoids.

Modern phytochemical and biological evaluations demonstrated that there are four primary bioactive constituents in *S. baicalensis*(4): baicalin (baicalein-7-*O*-glucuronide), baicalein (5,6,7-trihydoxyflavone), wogonoside (wogonin-7-*O*-glucuronide) and wogonin (5,7-dihydroxy-8-methoxyflavone) (Fig. 1A). These compounds are responsible for the observed pharmacological actions (5–7), such as anti-inflammation, reduction of total cholesterol level and blood pressure, and anti-HIV activities. Investigators have also reported the anticancer potential of *S. baicalensis*, as well as anti-chemotherapy-induced side effects (3,8,9).

Previous studies reported that the aglycons in *S. baicalensis* possessed remarkable anticancer effects (8,10). The two major aglycons in the herb, baicalein and wogonin, can be transformed from their glycosides, baicalin and wogonoside, respectively (8). Special TCM processing methods, such as high-temperature baking, steaming and fermentation, have been used in preparing Chinese herbal medicines, in order to reduce toxicity and adverse effects and to increase their effectiveness (11–14). However, these approaches are not likely to have a significant effect in promoting the transformation from flavonoid glycosides to aglycons.

Ginseng is another very commonly used herbal medicine worldwide. Previous studies observed that glycosidase including cellulase markedly catalyzed certain ginsenoside conversions to more bioactive compounds (15,16). However, an enzymatic catalyzing method has not been reported in preparation of *S. baicalensis*. In this study, using cellulase, we performed enzyme-catalyzed transformation of the herb to obtain aglycon-rich compounds, and their pharmacological activities were evaluated to support the effectiveness of this conversion. During the glycosidase-catalyzing of *S. baicalensis*, variable conditions (i.e., enzyme concentration, temperature, pH value) were used to obtain the optimal compound conversion rates. Then, different lengths of treatment time were used to prepare five distinct *S. baicalensis* extracts (SbEs). The anticancer potential of these five SbEs have subsequently been evaluated in connection with their aglycon contents using human HCT-116 colon cancer cells and MCF-7 breast cancer cells. The related anticancer mechanisms of action have also been explored.

## Materials and methods

### Chemicals and reagents

HPLC grade methanol, ethanol and acetonitrile were obtained from Tedia Co. (Fairfield, OH, USA). Milli-Q water was supplied by a Milli-Q Water Purification System (Millipore, MA, USA). Four flavonoid standards, i.e., baicalin, wogonoside, baicalein and wogonin, were obtained from the National Institute for Control of Pharmaceuticals and Biological Products (Beijing, China). All standards were of biochemical-reagent grade and at least 95% pure confirmed by HPLC. Cellulase (XW-G-F Cellulase, 140,000 U/g) was obtained from Novozymes Biologicals Co. (Shenyang, China). Trypsin, McCoy’s 5A and DMEM medium, fetal bovine serum (FBS), and penicillin-streptomycin solution (200X) were obtained from Mediatech (Herndon, VA, USA). A CellTiter 96 Aqueous One Solution Cell Proliferation Assay kit was obtained from Promega (Madison, WI, USA). Propidium iodide (PI) and RNase were obtained from Sigma (St. Louis, MO, USA). FITC Annexin V Apoptosis Detection kit was obtained from BD Biosciences (San Diego, CA, USA).

### Plant materials

The *S. baicalensis* root, collected from Gansu, China, was obtained from Tianyi Drugstore Co. (Huaian, China). The voucher samples were deposited at the Group of BioTCM and Biocatalysis, Faculty of Life Science and Chemical Engineering, Huaiyin Institute of Technology. Dried *S. baicalensis* roots were ground with a blade-mill (FW135 Medicine Mill, Nanjing, China) to obtain a relatively homogeneous drug powder, and then sieved through a 100-mesh (0.15 mm) screen. The powder was dried in a desiccator with silica gel at ambient temperature until constant weight before extraction.

### Determination of flavonoids

Ultraviolet (UV) spectra of flavonoids were recorded on a Shimadzu UV-2401PC UV-Vis spectrophotometer (Shimadzu, Japan). Quantitative determination was performed on an Agilent 1100 liquid chromatographic system (Agilent Technologies, CA, USA) consisted of a G1313A-ALS autosampler, a G1316A-Colume thermostated compartment, a G1311A-QuatPump and a G1379A degasser, a G1315B-DAD detector, and ChemStation software for peak identification and integration. The separation was carried out on an Agilent Zorbax Extend-C18 reversed-phase column (4.6×250 mm, 5.0 μm). A ternary isocratic solvent system of methanol-acetonitrile-water-acetic acid (18:25:57:0.2, v/v) was used. The flow rate was 0.8 ml/min and the detection wavelength was set to 275 nm. Temperature of column compartment was maintained at 30ºC. The injection volume was 10 μl. All tested solutions were filtered through a 0.2-μm membrane filter. The content of the constituents were calculated using calibration curves of four flavonoid standards.

### Condition tests for cellulase-catalyzed transformation

Cellulase was quantified accurately and dispersed in phosphoric buffer solution to obtain 20 ml of enzyme solution with a certain activity unit. One gram of herbal powder was added into the enzyme solution. After adjustment of the pH value, the flask containing the reaction mixture was fixed on a thermostatic water bath shaker (130 rpm) for a period of treatment time at a controlled temperature. After the treatment was finished, absolute ethanol was added to the reaction solution and the concentration of ethanol in the solution was 75% (v/v). The solution was sonicated in an ultrasonic bath (Kunshan Ultrasonic Instrument Co., China) for 30 min at 25ºC before the extract was filtered. The residues were ultrasonically extracted twice with 20 ml of 75% ethanol. All the filtrates were transferred into a 250 ml volumetric flask and adjusted to the volume with 75% ethanol. A 5.0 ml of extract solution was used for HPLC analysis. The solvent of the extract solution was evaporated under vacuum, and then the residue was completely dissolved in methanol. The following reaction conditions were tested: cellulase concentration (5–30 U/g), temperature (30–60ºC), pH value (4.0–5.0), and reaction time (0–10 h).

### Preparation of S. baicalensis extracts (SbEs)

After optimal conditions were obtained, a series SbE samples were prepared for biological evaluation. Briefly, 10 g of *S. baicalensis* root powder were added to 200 ml of reaction solution containing cellulase 20 U/g. The pH value was adjusted to 4.8. The temperature was set at 50ºC. The flask containing the reaction mixture was fixed on the shaker (130 rpm), and the reaction was conducted for 2, 4, 6 and 8 h. Subsequent treatment steps were the same as the above protocol. The filtrate collected was concentrated under vacuum (<60ºC) in a rotary evaporator to remove solvent. Then the extracts were lyophilized to obtain 2, 4, 6 and 8 h catalyzed SbE extracts: SbE2, SbE4, SbE6 and SbE8, respectively. Another control extract SbE0, which was without cellulase transformation, was also prepared. Before biological evaluation, the contents of the four flavonoids in SbEs were determined by HPLC.

### Cell culture

Two cell lines, HCT-116 human colorectal carcinoma cells and MCF-7 human breast cancer cells (ATCC, Manassas, VA, USA) were cultured in McCoy’s 5A and DMEM Media, respectively, supplemented with 10% fetal bovine serum and penicillin-streptomycin (50 U/ml). Cells were maintained at 37ºC in a humidified atmosphere, with 5% carbon dioxide and 95% air.

### Cell proliferation assay using modified trichrome strain (MTS) method

Cells were seeded in 96-well plates. After one day, various concentrations of SbEs (dissolved in DMSO) were added to the wells. The final concentration of DMSO was 0.5%. Controls were exposed to culture medium containing 0.5% DMSO without drugs. All experiments were performed in triplicate. Cell proliferation was evaluated using an MTS assay according to the manufacturer’s instructions. Briefly, at the end of the drug exposure period, the medium was replaced with 100 μl of fresh medium and 20 μl of MTS reagent (CellTiter 96 Aqueous Solution) in each well, and the plate was returned the incubator for 1–2 h. A 60-μl aliquot of medium from each well was transferred to an ELSA 96-well plate and its absorbance at 490 nm was recorded. Results were expressed as a percentage of the control (DMSO vehicles set at 100%).

### Cell cycle analysis of HCT-116 cells after staining with propidium iodide

To further evaluate the effects of SbEs on anticancer potential, we performed cell cycle analysis of HCT-116 treated with different concentrations of SbE0 and SbE8. HCT-116 cells were seeded in 24-well tissue culture plates. On the second day, the medium was changed, and cells were treated with different concentrations of SbE0 or SbE8 for 48 h. After harvesting, the cells were fixed gently by adding 80% ethanol and placing them in a freezer for 2 h. They were then treated with 0.25% Triton X-100 for 5 min in an ice bath. The cells were resuspended in 300 μl of PBS containing 40 μg/ml propidium iodide (PI) and 0.1 mg/ml RNase. Cells were incubated in a dark room for 20 min at room temperature, and then subjected to cell cycle analysis using a FACScan flow cytometer (Becton Dickinson, Mountain View, CA, USA) and FlowJo 10.0.4 software (Tree Star, Ashland, OR, USA). For each measurement, at least 20,000 cells were counted.

### Apoptosis analysis of HCT-116 cells after staining by Annexin V-FITC/propidium iodide

Cells were seeded in 24-well tissue culture plates. After one day, the medium was changed, and SbE0, SbE4 and SbE8 were added. After treatment for 48 h, cells floating in the medium were collected. The adherent cells were detached with 0.25% trypsin. Then, culture medium containing FBS (and floating cells) was added to inactivate trypsin. After pipetting gently, the cells were centrifuged for 5 min at 1500 × g. The supernatant was removed and cells were stained with Annexin V-FITC and propidium iodide (PI) according to the manufacturer’s instructions. Untreated cells were used as a control for double staining. The cells were analyzed immediately after staining using a FACScan flow cytometer. For each measurement, at least 20,000 cells were counted.

### Statistical analysis

Cell proliferation, and flow cytometry experiments were performed in triplicate. The data are presented as mean ± standard error (SE). A one-way ANOVA determined whether the results had statistical significance. In some cases, a Student’s t-test was used for comparing two groups. The level of statistical significance was set at P<0.05.

## Results

### Ultraviolet spectra and HPLC chromatogram of four flavonoids

Fig. 1A shows the chemical structures of four flavonoids (baicalin, wogonoside, baicalein and wogonin) identified from *S. baicalensis*. Ultraviolet spectroscopy of these four flavonoids is shown in Fig. 1B. The maximum absorption wavelength (*λ*_max_) of the four flavonoids was 277.5, 275.5, 275.0 and 275.5 nm, respectively. Thus, for the HPLC analysis, 275 nm was a suitable wavelength for the determination of the four flavonoids. Fig. 1C is the HPLC chromatogram recorded at 275 nm. The four flavonoids were well separated under the established HPLC condition, and this HPLC method can be utilized to accurately measure these compound contents in the test samples.

### Optimization of conditions for cellulase catalyzed deglycosylation

**Figure 2:**
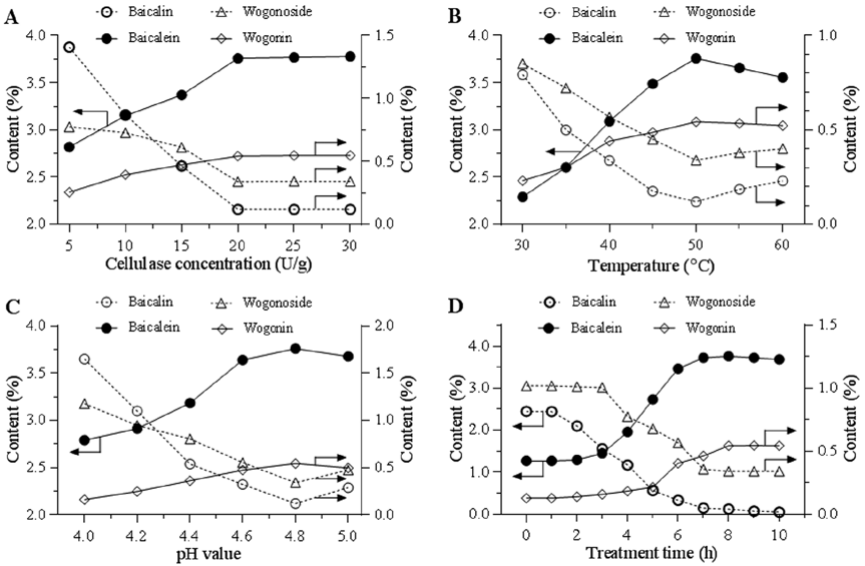
Determination of the optimal conditions of cellulase in catalyzing flavonoids in *S. baicalensis*. Cellulase concentration at 20 U/g (A), temperature at 50ºC (B), and pH value at 4.8 (C) were chosen for better catalyzing activity. (D) At the above conditions, the length of treatment time significantly affected the conversion rate of the flavonoids.

To determine the optimal conditions of cellulase in catalyzing flavonoids from *S. baicalensis*, variable conditions were tested. Fig. 2A indicates the effects of cellulase concentrations from 5–30 U/g. Along with an increase of the enzyme concentration, an increase of the two aglycon contents and a decrease of the two glycoside contents were observed. Using the selected cellulase concentration of 20 U/g, the effect of temperature (30–60ºC) on transformation was tested. Fig. 2B indicates that 50ºC was the optimal reaction temperature. Using the selected cellulase concentration and temperature, Fig. 2C indicates the effects of pH value from 4.0–5.0, and a pH of 4.8 was selected for further evaluation. Fig. 2D shows the effects of treatment time from 0 to 10 h. The content curves of the two aglycons and two glycosides were obtained and they clearly indicate that treatment time significantly affected the flavonoid conversion. Overall, the optimized catalyzing conditions are: 20 U/g of enzyme, pH 4.8, treatment at 50ºC for 8 h.

### Preparation of cellulase catalyzed S. baicalensis extracts (SbEs) for bioactivity assay

**Figure 3:**
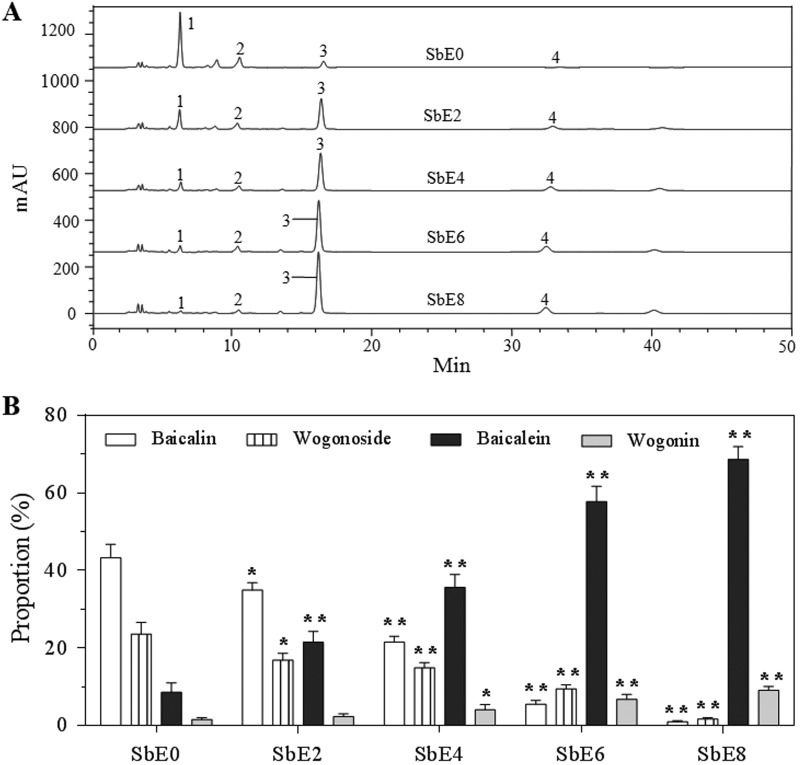
Determination of four flavonoids in cellulase catalyzed *S. baicalensis* extracts. Different treatment times affecting flavonoid contents. (A) HPLC chromatograms of five SbE samples (SbE0, SbE2, SbE4, SbE6 and SbE8) recorded at 275 nm. Flavonoid peaks: 1, baicalin; 2, wogonoside; 3, baicalein; 4, wogonin. (B) Variable proportion of the four flavonoids in the five SbEs. ^*^p<0.05; ^**^p<0.01 vs. SbE0.

Based on Fig. 2D observation, at fixed conditions (cellulase 20 U/g, pH 4.8 and 50ºC), different lengths of time were used to treat *S. baicalensis*. Fig. 3A shows HPLC chromatograms of five SbE sample treatments for 0, 2, 4, 6 and 8 h (SbE0, SbE2, SbE4, SbE6 and SbE8). Significantly different proportions of the four flavonoids in the five SbEs are shown in Fig. 3B. For SbE0, the content of the two glycosides was highest, and the content of the two aglycons was the lowest. However, for SbE8, the content of the two glycosides was the lowest, and the content of the two aglycons was the highest. The proportion of baicalin, wogonoside, baicalein and wogonin, was 43.3, 23.6, 8.5 and 1.6% in SbE0; 34.9, 16.8, 21.5 and 2.3% in SbE2; 21.5, 14.9, 35.6 and 4.1% in SbE4; 5.5, 9.4, 57.7 and 6.7% in SbE6; and 1.0, 1.7, 68.6 and 9.1% in SbE8, respectively. The results indicate that the proportion of the four flavonoids in the SbEs was significantly changed after cellulase treatment.

### Antiproliferative effect of five SbEs on HCT-116 and MCF-7 cells

**Figure 4:**
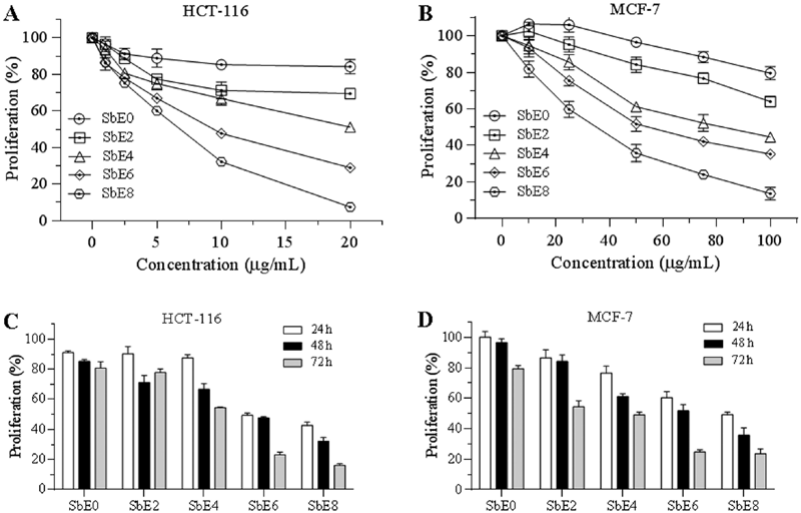
Concentration- and time-dependent effects of SbEs on proliferation of human HCT-116 colon cancer cells and MCF-7 breast cancer cells. (A) Effects of five SbEs at different concentrations (1.0, 2.5, 5.0, 10.0 and 20.0 μg/ml) on HCT-116 cells, and (B) at different concentrations (10, 25, 50, 75 and 100 μg/ml) on MCF-7 cells are shown. (C) Effects of 10 μg/ml SbEs on HCT-116 cells, and (D) 50 μg/ml SbEs on MCF-7 cells at different time points (24, 48 and 72 h) are presented.

Effects of cellulase-catalyzed extracts on cancer cell growth inhibition were assayed. As shown in Fig. 4, after treatment with 20 μg/ml for 48 h, SbE0 inhibited HCT-116 cell growth by 15.9%, while SbE8 inhibited cell growth by 92.6%. Antiproliferative potential of other SbEs were between SbE0 and SbE8 (Fig. 4A). Sensitivity of MCF-7 cells by SbEs was lower than that of HCT-116. At 100 μg/ml, SbE0 inhibited MCF-7 cell growth by 20.5%, while SbE2, SbE4, SbE6 and SbE8 inhibited cell growth by 26.1, 55.4, 64.8 and 86.4%, respectively (Fig. 4B). Of note, at some lower concentrations, SbE0 and SbE2 actually improved MCF-7 cell growth. The IC_50_ (50% inhibitory concentration) of SbE8 for HCT-116 and MCF-7 cells was ~5 and 30 μg/ml, respectively. Incubation time also influenced antiproliferative effects of SbEs on cancer cells. In treatment with 10 μg/ml of SbE8 for 24, 48 and 72 h, HCT-116 cell growth was inhibited by 57, 68 and 84%, respectively (Fig. 4C). Similar results were also observed on MCF-7 cells (Fig. 4D), suggesting that SbEs inhibit cancer cell proliferation in a time-dependent manner. In addition, SbE8 had the strongest antiproliferative effects on both cancer cell lines. These results demonstrated that 8 h of cellulase catalyzing significantly increased the antiproliferative potential of *S. baicalensis*.

### Effect of SbE0 and SbE8 on HCT-116 cell cycle

**Figure 5:**
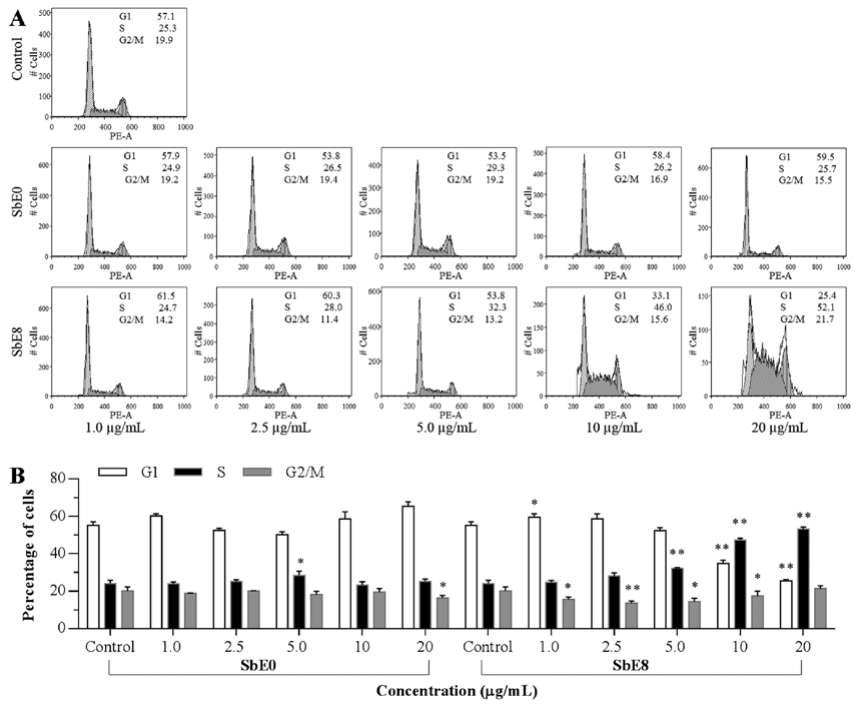
Cell cycle analysis of HCT-116 cells using flow cytometry after staining with propidium iodide/RNase. HCT-116 cells are treated with SbE0 or SbE8 at variable concentrations (1, 2.5, 5, 10 and 20 μg/ml) for 48 h. (A) Representative histograms of the DNA content in each experimental group. (B) Percentage of each cell cycle phase with various treatments or with control. Data are presented as the mean ± standard error of triplicate experiments. ^*^p<0.05, ^**^p<0.01 vs. control.

To explore the potential mechanism by which cellulase-catalyzed extract inhibits HCT-116 cell growth, the cell cycle profile was assayed by flow cytometry. As shown in Fig. 5, compared to the control, SbE0 did not change the cell cycle profile obviously, whereas significant changes were observed with SbE8. Although treatment with SbE8 at 1 and 2.5 μg/ml did not change the cell cycle profile, when the treatment concentration was increased, the cell cycle profile was changed. Compared to the control (57.1% of G-phase, 25.3% of S-phase, and 19.9% of G2/M-phase), the percentage of G1-, S- and G2/M-phase cells after treatment with SbE8 for 48 h were 33.1, 46.0 and 15.6% at 10 μg/ml; and 25.4, 52.1 and 21.7% at 20 μg/ml, respectively. These results indicate that SbE8 significantly induced cell cycle arrest in the S-phase.

### Apoptotic effect of SbE0, SbE4 and SbE8 on HCT-116 cells

**Figure 6:**
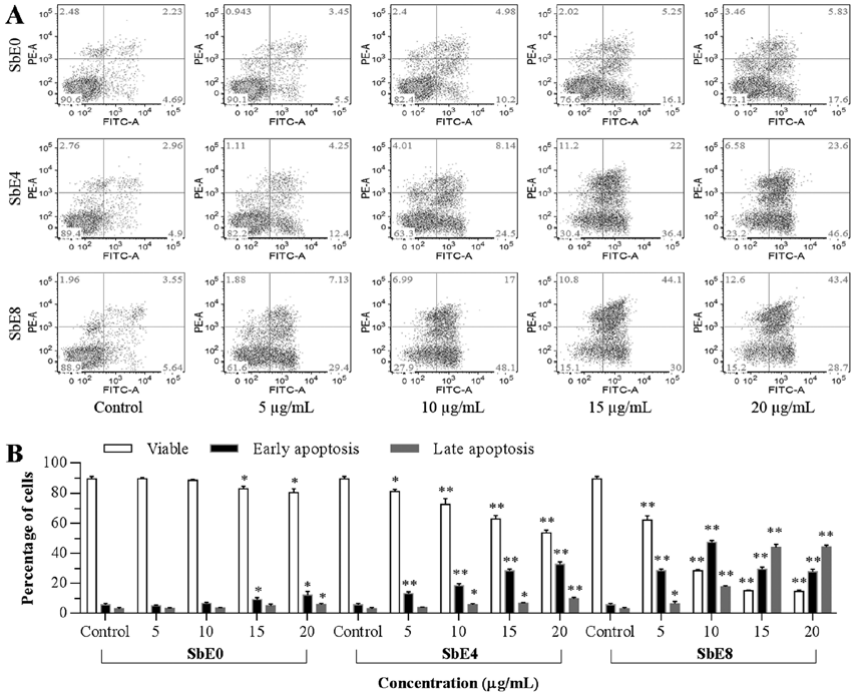
Apoptosis assay using flow cytometry after staining with FITC-Annexin V/propidium iodide. HCT-116 cells are treated with SbE0, SbE4 or SbE8 at variable concentrations (5, 10, 15 and 20 μg/ml) for 48 h. (A) Representative scatter plots of PI (y-axis) vs. Annexin V (x-axis). (B) Percentage of viable, early apoptotic and late apoptotic cells. Data are presented as the mean ± standard error of triplicate experiments. ^*^p<0.05, ^**^p<0.01 vs. control.

The cell proliferation assay results suggested that the cancer cell growth inhibitory capacity of SbEs is related to the cellulase pretreatment time (Fig. 4). To further explore the mechanisms of actions of SbEs, we carried out an apoptotic assay. As shown in Fig. 6, compared to the control (early apoptosis 4.7–5.6%, late apoptosis 2.2–3.6%), after 48 h of treatment with 5 and 10 μg/ml of extracts, SbE0 did not increase early and late apoptosis while SbE4 moderately increased the early apoptosis to 17.6%, whereas SbE8 quickly increased the early apoptosis to 48.1%. At the same time, we observed SbE4 and SbE8 significantly increased late apoptosis. This result suggests that the antiproliferative effect of SbE4 and SbE8 was mediated by the induction of apoptosis, and induced apoptosis in a dose-dependent manner. Furthermore, it was shown that apoptotic induction potential was positively correlated with the cellulase treatment time.

## Discussion

The aims of processing Chinese medicinal herbs are to reduce or even eliminate the botanically-related toxicity and side effects and/or to elevate their therapeutic activities. In addition, some processing procedures can modify a given herb’s medicinal character based on the TCM theory, making it more compatible with other herbs for a TCM formulation (17,18). In our previous studies, we observed that heat-processed American ginseng and *Panax notoginseng* converted their ginsenoside profile and increased the anticancer potential. However, the steaming method did not markedly affect the *S. baicalensis* composition profile, and more importantly, the high temperature could even degrade some flavonoid structures (19,20).

In the present study, we utilized cellulase to catalyze flavonoids in *S. baicalensis*. Our HPLC analysis showed that, at optimized conditions obtained in our study, there was a very significant transformation from two glycosides, baicalin and wogonoside, to two aglycons, baicalein and wogonin, respectively. The anticancer potential was further tested using five different SbE extracts. These five extracts received variable lengths of treatment time with cellulase, and different levels of baicalein and wogonin were shown in HPLC chromatograms (Fig. 3). Subsequently, human colon cancer and breast cancer cells were used to evaluate antiproliferative potential of these five SbEs, and our data showed that the higher the aglycon content, the stronger the antiproliferation activities (Fig. 4). This observation is consistent with our previous study (8).

Many studies have demonstrated anticancer effects of baicalein (10,21,22). We also observed that baicalein could protect against chemotherapeutic agent-induced cardiotoxicity by attenuation of oxidant injury and JNK activation (9). To further increase the bioactivity of a botanical compound, we previously prepared derivatives of protopanaxadiol (PPD), a ginseng compound, for enhanced anticancer activity (23). Attempts have also been made for novel synthetic baicalein derivatives against human tumor cells (24), and the structure modification could be one of interesting directions in future baicalein research.

Compared to numerous baicalein anticancer investigations, evaluation of wogonin has received less attention. Recently, however, data showed that both baicalein and wogonin had antitumor actions in human HT-29 human colorectal cancer cells (25). The functional role of wogonin in anti-angiogenesis has also been reported. Wogonin suppressed IL-6-induced vascular endothelial growth factor (VEGF) by modulating the IL-6R/JAK1/STAT3 signaling pathway, suggesting that the compound provided a therapeutic option in the treatment of the related pathological angiogenesis (26). In our future studies, it would be helpful to compare the bioactivity of baicalein and wogonin using the same experimental conditions.

Similar to most Chinese herbal medicines, *S. baicalensis* is ingested orally. In the present study, we evaluated the herbal anticancer potential using *in vitro* human cancer cell lines for initial antiproliferation comparison of five SbEs with variable aglycon concentrations. The next step should be to use *in vivo* cancer models to further verify our observations. For *in vivo* long-term antitumor observation, it would be desirable to mix the botanical extract or compound(s) with animal chow, a safe and practical method for drug delivery.

Two important issues are related to herbal oral administration. First, the test compound bioavailability and pharmacokinetic data should be obtained, which have been examined previously using both baicalin and baicalein (27,28). Secondly, intestinal microbiota play a key role in botanical compound biotransformation (29,30). As a result, the bioactivity of a given compound could be increased or reduced due to the effects of intestinal microbiota. We previously observed that after oral ginseng administration, ginseng saponins were metabolized extensively by intestinal microbiota (14,31,32). Thus, in understanding the pharmacological actions of *S. baicalensis*, a great deal of attention should be paid to compound metabolites via gut microbiome.

Cellulase refers to a group of enzymes which, acting together, hydrolyze cellulose and other compounds. The cellulase contain different enzymes, such as endoglucanase, cellobiohydrolase, β-glucosidase and other glycosidases (33,34). In future studies, which enzyme in cellulase played the key role in the conversion from glycoside to aglycon remains to be elucidated.

In conclusion, for the first time, we utilized cellulase, a group of glycosidase, to convert two glycosides in *S. baicalensis* into two aglycons, baicalein and wogonin. Using optimal conditions identified in our study, glycosidase catalyzing very markedly increased the content of the two aglycons. Comparison studies using five distinct SbEs showed that the higher the aglycons, the better the anticancer activity. Further studies, including the use of *in vivo* animal cancer models, should be conducted. However, the present study demonstrated that using glycosidase to catalyze *S. baicalensis* offers a promising approach to increasing the herb’s anticancer potential.
